# The Impact of Complex Loadings on the Structure of the L2-L3 Intervertebral Disc in a Sheep Spine Cadaver Model: A Biomechanical and Histological Evaluation

**DOI:** 10.7759/cureus.51941

**Published:** 2024-01-09

**Authors:** Akın Öztürk, Ahmet Karakaşlı, Güven Erbil, Mehmet Burak Gökgöz, Furkan Yapici

**Affiliations:** 1 Orthopaedics and Traumatology, Mengücek Gazi Hospital, Erzincan, TUR; 2 Orthopaedics and Traumatology, Dokuz Eylül University Faculty of Medicine, İzmir, TUR; 3 Histology and Embryology, Kyrenia University Faculty of Medicine, Girne, CYP; 4 Orthopaedics and Traumatology, Erzincan University Faculty of Medicine, Erzincan, TUR

**Keywords:** lumbar disc hernia, spine, orthopedics, multidisciplinary study, histological study, biomechanical study, complex loadings, cadaver model, sheep spine, intervertebral disc

## Abstract

Background

The human vertebral column generates movements under versatile, dynamic loads. Understanding how the spine reacts to these movements and loads is crucial for developing new spine implants and surgical treatments for intervertebral disc injuries. Mechanically uni-axial compression models have been extensively studied. However, the spine's daily loading is not limited to compression, so it is crucial to measure its behavior in all movements (flexion-extension, rotation, and axial compression).

Methods

This study utilized L1-L5 segments from 19 healthy adult sheep spines. The L2-L3 disc of the first spine underwent only histological evaluation without biomechanical testing to define basic histological parameters. The remaining 18 were divided into three groups of six and subjected to biomechanical tests. Different mechanisms for three groups of spinal segments were prepared, and tests were performed on Shimadzu AG-IS 10 KN (Universal Drawing Press, Kyoto, Japan). An axial load (800 N) was applied to the first group, an axial load with 15 degrees of flexion to the second group, and an axial load with 10 degrees of rotation plus 15 degrees of flexion to the third group. A biomechanical evaluation of the maximum elongation amounts (MEAs) was performed and compared between the groups. Then, the L2-L3 discs were removed from the sheep spines, and a histological examination of the discs was conducted using Hematoxylin-Eosin (HE), Alcian Blue (AB), and Masson's Trichrome (MT) staining.

Results

The mean MEA ± Standard Deviation (Range) was 1.39 ± 0.38 (0.91-1.94) for Group 1, 2.02 ± 0.75 (0.91-3.01) for Group 2, and 2.47 ± 1.09 (0.64-3.9) for Group 3. Biomechanically, although MEAs increased from Group 1 to Group 3 (meaning that the mean MEAs increased as the number of types of applied force increased), there was no statistically significant difference between the groups regarding the MEAs (P = 0.092). Histologically, no significant differences were observed between all groups after HE staining. In all groups, hypercellularity, edema in the connective tissue, separation between tissue layers, delamination, and signs of swelling and necrosis in the cells were observed similarly. For the AB staining, there was a decrease in the glycosaminoglycan (GAG) structure in the tissue samples compared to the control tissue, but no significant differences were observed between the groups. However, it was observed that the stratification in Group 3 was slightly more deteriorated than in the other groups. For the MT staining, collagen structure deterioration was observed in all groups. It was observed that the amount of collagen was significantly reduced compared to the control tissue.

Conclusion

As a result, when the axial load is applied biomechanically, there is more displacement of the vertebral discs in Group 3 with multidimensional movements. Furthermore, histological studies revealed deterioration between tissue layers when exposed to complex movements, and the degradation of stratification in group 3 compared to other loading combinations in groups 2 and 3 may indicate the role of complex loads in the formation of disc herniation.

## Introduction

Low back pain is a common problem in society and contributes to workforce loss. Many studies point to the high frequency of back complaints in the community. The lifetime prevalence of low back pain is estimated at 80%, with annual hospital admission rates of 15% in the adult population [1]. A 2008 global review encompassing 165 studies from 54 countries reported a mean point prevalence of 18.3% [2]. This health problem is among the first diseases regarding social activity and workforce loss. According to studies conducted in the United States, 60%-90% of the adult population faces chronic low back pain at some point in their lives, and this condition ranks second among the causes of workforce loss after upper respiratory tract diseases [3,4]. Also, in the United States, back pain is the most common reason for limitation of activity in people under 45 years of age, the fifth most common reason for hospital admission, and the third most common reason for surgery [5]. The data in other Western countries is similar. In the United Kingdom, for example, it was estimated that the most significant cause of absence from work in 1988-89 was low back pain, accounting for approximately 12.5% of all sick days [6]. Again, it is estimated that people with chronic low back pain cost roughly $11 billion per year in the United Kingdom [7] and almost $5 billion in the Netherlands [8]. Although this problem is generally seen between the ages of 40 and 50, studies argue that it occurs more frequently, especially in those working in the industrial and service sectors [9-12].

Low back pain is a symptom. The most common form is vague lower back pain. This term is used when the pathoanatomical cause of the pain cannot be determined. Since pain in the lumbar region can arise from all anatomical structures, it cannot be well localized by the doctor and the patient [13]. Except for specific pain foci, such as intervertebral discs and facet joints, clinical tests cannot reliably indicate the source of pain [14].

Clinical studies show that the source of low back pain is due to intervertebral disc pathologies at a rate of up to 39%. When considered together with the subgroups of intervertebral disc pathologies, lumbar disc herniation (LDH) and degenerative disc disease take the lead [13]. Although the spine is subject to normal age-related processes, it is also subject to specific disorders, such as degenerative disc disease and disc herniation. With the imaging techniques developed in recent years, LDH can also be detected in non-symptomatic cases [15].

The intervertebral disc is a tissue with a heterogeneous character consisting of the nucleus pulposus (NP), annulus fibrosus (AF), and endplate substructures. These tissues work together to allow the spine flexibility, support, and transfer large multidirectional loads. LDH is a herniated NP pathology that initially presents with increasing back pain, followed by sudden hip and leg pain. Quality of life decreases in patients who most commonly present with complaints of low back pain, leg pain, neurogenic claudication, and sphincter defects. Patients' symptoms depend on posture, increasing with extension and weight-bearing positions and decreasing with flexion or unloaded postures [3]. The human spine provides movements under multidirectional, dynamic loads. Understanding how the spine responds to these movements and loads is critical to developing new spinal implants and surgical treatments for intervertebral disc injuries. To experimentally measure this response in vitro, excised human spinal segments have been extensively studied in mechanically uniaxial compression models to obtain the viscoelastic and poroelastic properties of the disc [16]. However, the loading of the spine in daily life is not limited to compression, so it is essential to measure its behavior in all movements (flexion-extension, rotation, and axial compression).

In the current literature reviews, although there are studies on different loading models that the spine is exposed to daily, there are deficiencies in lumbar intervertebral disc histology after these loads. In our research, in coordination with Dokuz Eylül University Health Sciences Institute Biomechanics Department and Dokuz Eylül University Faculty of Medicine Department of Histology and Embryology, sheep vertebrae were examined in three groups, respectively, under axial loading only, under axial loading in flexion, and under axial loading with rotation in flexion. We aimed to examine biomechanically and histologically and to investigate and document possible differences in the lumbar intervertebral disc structure as a result of complex loads.

## Materials and methods

In this study, L1-L5 segments and L2-L3 discs of a total of 19 full-grown healthy sheep vertebrae were used. After supplying the spines, they were kept at minus 18 degrees until the study was to be carried out. Before the analysis, the spines were removed from the refrigerator the night before to thaw at room temperature. One of the 19 lumbar vertebra segments was separated from the others without biomechanical testing and only to see the standard histological structure of the L2-L3 intervertebral disc (IVD). The remaining 18 vertebrae were randomly divided into three groups, with six vertebrae in each group. After preparing as described in the preparation section before the biomechanical test, biomechanical tests were performed in the laboratory of the Dokuz Eylül University Health Sciences Institute Biomechanics Department on L1-L5 vertebra segments. Histological examination was performed on the same specimens (L2-L3 IVDs of the regarding L1-L5 vertebra segments) in the laboratory of the Dokuz Eylül University Faculty of Medicine, Histology, and Embryology Department, as described in the preparation section before the histological examination.

Preparation before biomechanical tests

**Figure 1:**
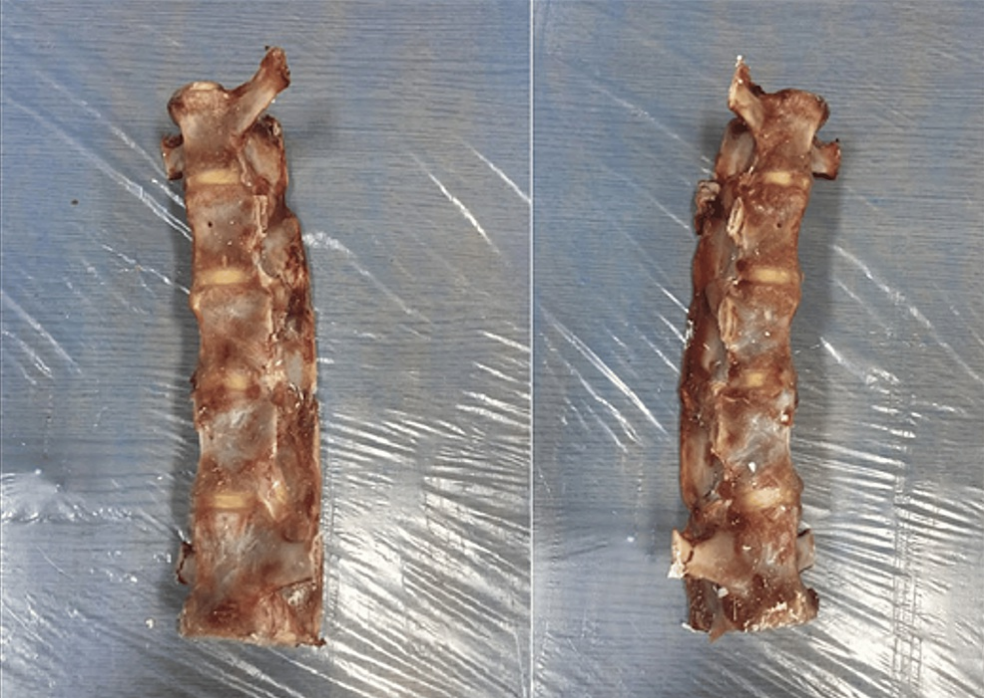
The sheep spine segment was obtained by cutting from the upper disc of the L1 vertebra and the lower disc of the L5 vertebra.

**Figure 2:**
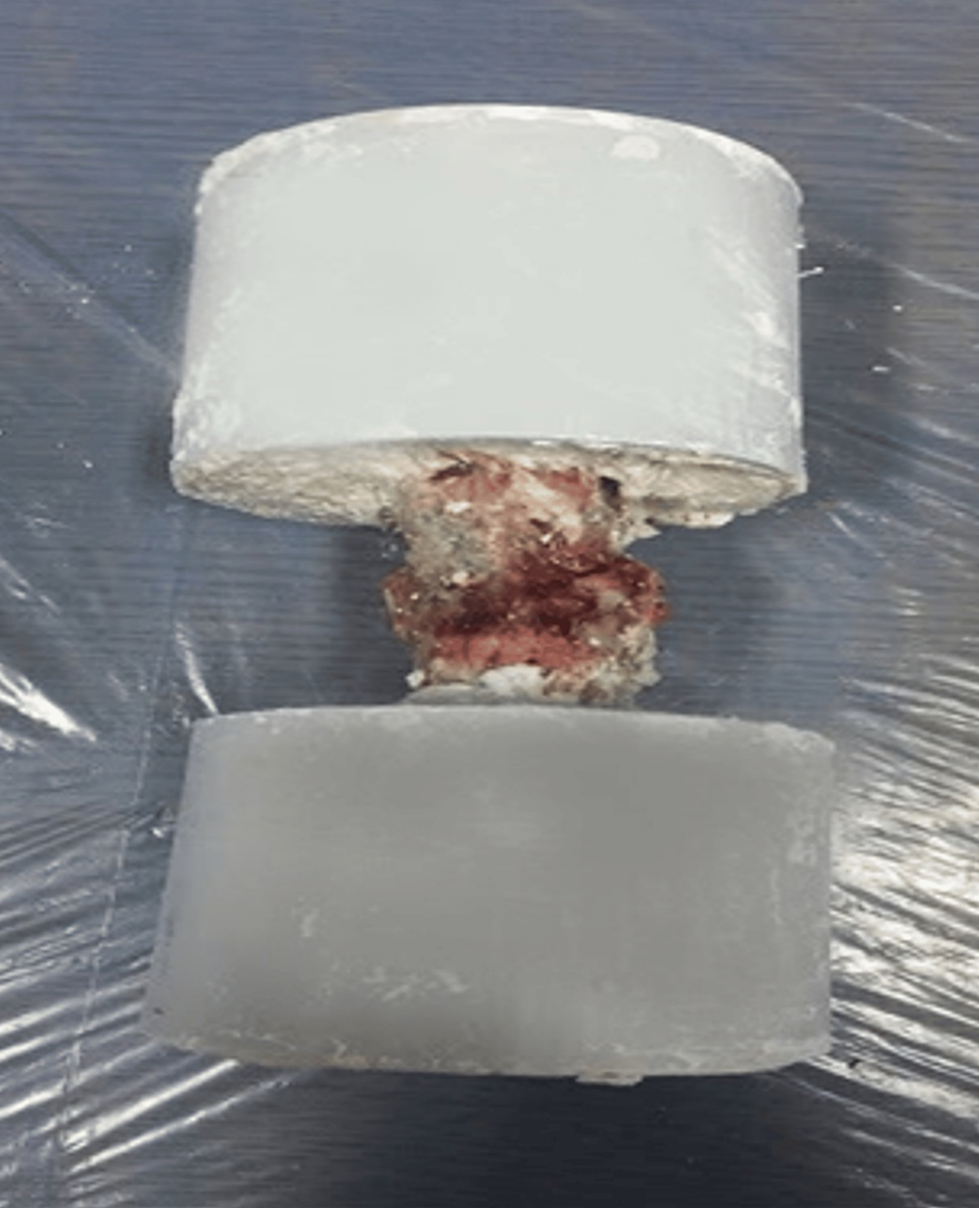
The L1-L5 lumbar vertebra segment of sheep is potted with cement.

The lumbar segments of all sheep vertebrae were separated from the upper disc of the L1 vertebra and the lower disc of the L5 vertebra to obtain L1-L5 segments. The transverse processes of these vertebral segments were cut with appropriate cutters. Only the uppermost L1 vertebral transverse processes and the lowermost L5 vertebral transverse processes were cut to leave approximately 2 cm longer to increase the stability against the rotation moment in the cement (Figure 1). In order to connect these created lumbar vertebra segments to the biomechanical test device, the potting process was performed using cement from the upper and lower parts (Figure 2). While this process was being performed, the potting process was performed so that the entire L1 vertebra and the proximal half of the L2 vertebra remained in the cement on the upper side, and the distal half of the L4 vertebra and the entire L5 vertebra remained in the cement on the lower side. In this way, all vertebral segments' L2-L3 and L3-L4 discs were left exposed. The same apparatus mentioned above was prepared for each spine, and biomechanical tests were applied to the L1-L5 segments in three different groups. Using its original computer program, the tests were performed on the Shimadzu AG-IS 10 KN (Universal Drawer Press, Kyoto, Japan) device in the biomechanics laboratory.

Biomechanical tests

Group 1: Axial Load Only

**Figure 3:**
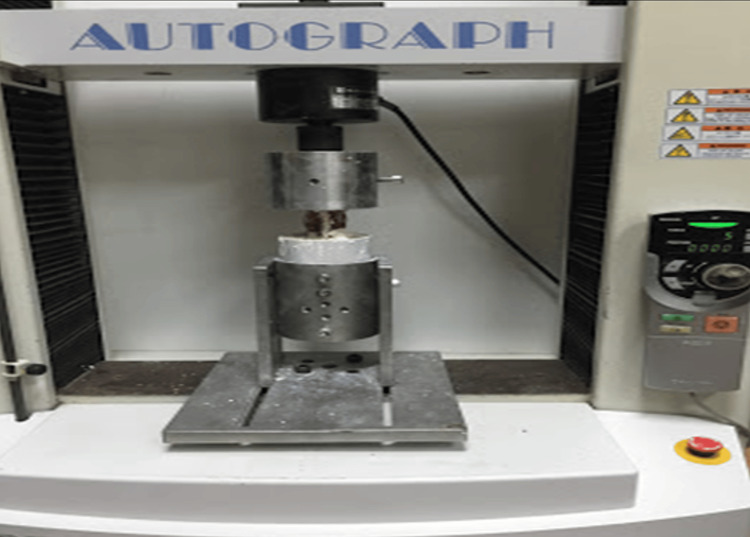
The L1-L5 segment of the sheep spine was subjected to axial loading only.

The pre-potted sheep L1-L5 vertebral segments were placed on the biomechanical testing device with the L1-L2 vertebrae on top and the L4-L5 vertebrae on the bottom. After the load transmitter arm of the device was contacted from the top and bottom, the load values were reset and made ready for the loading test (Figure 3).

Group 2: Axial Load With Flexion

**Figure 4:**
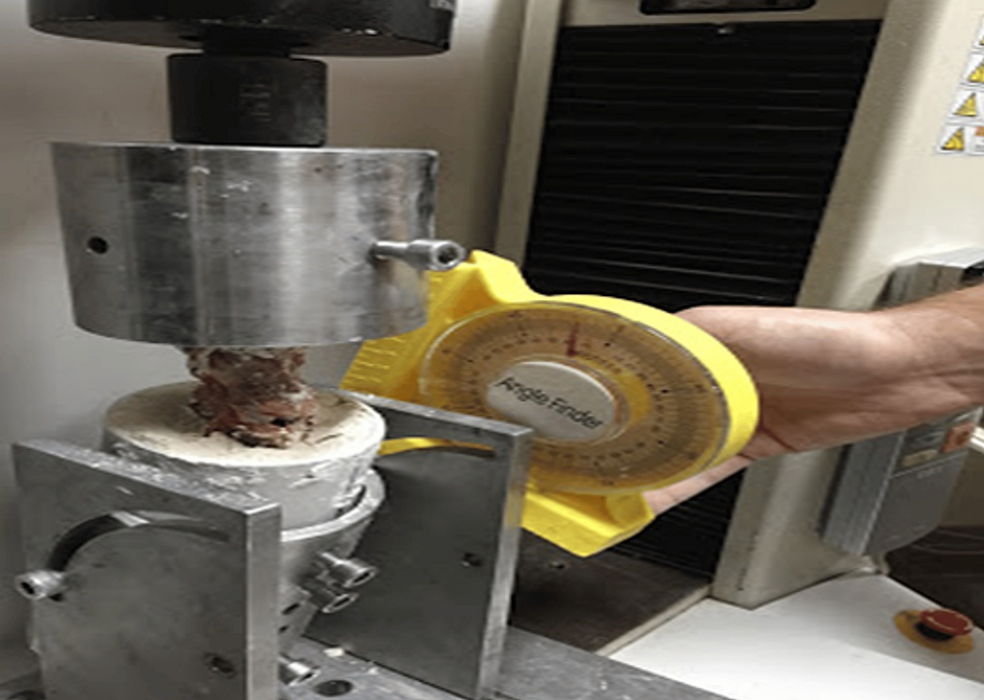
The L1-L5 segment of the sheep was spine subjected to axial loading with 15 degrees of flexion.

Six sheep spinal lumbar L1-L5 segments were placed on the device with approximately 15 degrees of flexion. Connection mechanisms were used to create 15 degrees of flexion and place it on the device. Once the device's load transmitter arm was contacted from the top and bottom, the load values were reset and made ready for the load test (Figure 4).

Group 3: Axial Load With Flexion and Rotation

**Figure 5:**
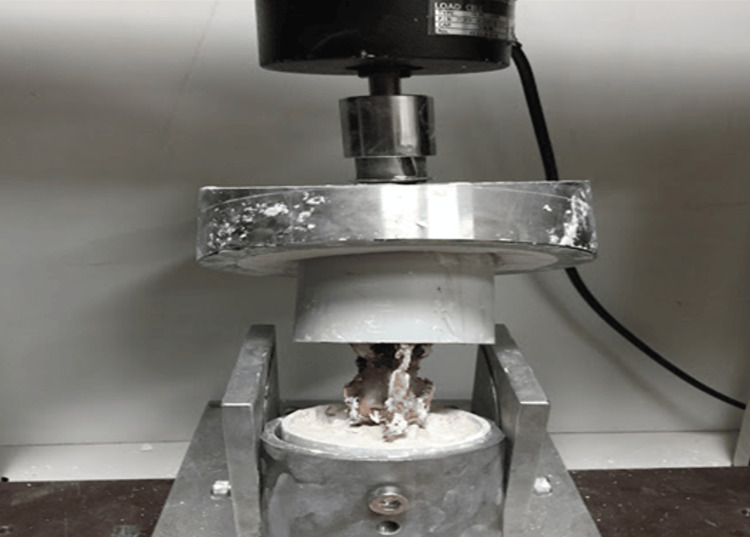
The L1-L5 segment of the sheep spine was subjected to axial loading with 15 degrees of flexion and 10 degrees of rotation.

Axial load was applied to six sheep L1-L5 spinal segments using a specially designed device that can be connected to a biomechanical testing device and can simultaneously apply 15 degrees of flexion and 10 degrees of rotation due to its design (Figure 5). After the load transmitter arm of the device was contacted from the top and bottom, the load values were reset and made ready for the load test.

Biomechanical test protocol

A load of 800 Newtons was applied at a 30 mm/min speed for all three groups to the L1-L5 segments of the sheep lumbar vertebrae, which were placed on the device as previously described to determine the maximum elongation amounts at yield. The device's original software generated the results showing the amount of force applied to each specimen and displacement. For biomechanical tests, the Berger-Roscher N et al. study protocol in 2017 was taken as a basis [17].

Preparation before histological tests

A total of 19 sheep’s lumbar segment's L2-L3 discs (18 biomechanically tested discs and one untested disc) were cut and sampled. To prepare vertebral disc samples for histological examination, initiate the process by immersing them in 10% neutral buffered formalin for 48 hours at room temperature. Following fixation, meticulously wash the tissues in running water overnight to eliminate excess fixative. Subsequently, proceed with dehydration by either employing a tissue processing machine or conducting manual dehydration with escalating concentrations of ethyl alcohol. Commence with 70% ethanol for 20 minutes, 80% ethanol for 20 minutes, and culminate with two changes of 96% ethanol, each for a duration of 20 minutes. The samples were kept in xylol twice for 30 minutes each. After applying paraffin twice, one hour each, in a 60˚C oven for two hours and ensuring immersion with paraffin, the tissues were embedded in paraffin blocks. 5μ-thick sections were taken using a rotary microtome (RM 2255, Leica).

Hematoxylin and eosin staining protocol

In the histological protocol for Hematoxylin and Eosin (HE) staining of vertebral disc tissue, the sections were initially deparaffinized by incubating in an oven at 56˚C for four hours. Following deparaffinization, the sections underwent a series of solvent exposures, starting with two rounds of Xylene for 2 minutes each, followed by sequential immersion in 100% ethanol (2 minutes), 95% ethanol (2 minutes), and a water wash (2 minutes). The subsequent staining steps included immersion in Hematoxylin (05-06002L, BioOptica, Milan, Italy) for 3 minutes, followed by a water wash for 1 minute. After Eosin (05-10003L, BioOptica, Milan, Italy) staining, the sections were subjected to a series of ethanol solutions (45 seconds in 95% ethanol, 1 minute in 100% ethanol, and another 1 minute in 100% ethanol) before two rounds of Xylene exposure for 2 minutes each. The final step involved covering the sections with a coverslip. HE-stained slides were examined under a light microscope. The structural properties of the tissues were evaluated by comparing them with the properties of the control tissue in terms of tissue integrity disruption and delamination.

Masson's trichrome staining protocol

The 5 µ tissue sections of formalin-fixed tissues were utilized for Masson's trichrome (MT) staining (5022, GBL, Istanbul, Turkey). The sections were initially deparaffinized through a series of xylene and ethanol treatments, followed by a re-fixation step in Bouins fluid at 56°C for one hour in the case of formalin-fixed samples. Subsequently, thorough washing with tap water was conducted to eliminate any residual yellow coloration. The staining process involved the use of Weigert’s iron hematoxylin or an equivalent nuclear stain, followed by a sequence of solutions labeled A, B, and C, each with specific staining durations according to the manufacturer’s recommendation. After staining, the sections underwent thorough rinsing with distilled water. Ethanol was then employed for dehydration, and xylene was used to clear the samples before mounting them with a resinous medium. MT-staining is used to visualize connective tissues, particularly collagen, in tissue sections. MT-stained slides were examined under a light microscope. The staining intensity, collagen amount, collagen structure, and organization features of the tissues were evaluated by comparing them with the features of the control tissue.

Alcian blue staining protocol

For deparaffinization, the sections were kept in an oven at 56˚C for two hours and held in three different xylols, one of which was in the oven. It was then passed through a series of decreasing alcohols. After being rinsed with distilled water, it was treated with alcian blue for 30 minutes. Then, it was treated with neutral red for five minutes without washing. After staining, the sections were washed with distilled water and passed through an increasing alcohol series. After clarification with xylol, it was sealed with a cover slip. AB staining is used to visualize GAG in tissue sections. AB-stained slides were examined under a light microscope. The staining intensity, structure, and organization properties of the tissues were evaluated by comparing them with the properties of the control tissue.

Statistical analysis

After all biomechanical tests were completed, the maximum elongation amounts (MEAs) obtained for 18 vertebrae in three different groups after static loading were recorded for data analysis. Statistical analysis was performed using IBM Corp. Released 2013. IBM SPSS Statistics for Windows, Version 22.0. Armonk, NY: IBM Corp. The distribution normality was tested with the Shapiro-Wilk test. The homogeneity of distribution between groups was tested with the Levene test. In evaluating the study data, in addition to descriptive statistical methods (mean, standard deviation), the ANOVA test was used to compare the quantitative data between the groups and to determine the difference. The results were evaluated at the 95% confidence interval, and the significance level was p < 0.05.

## Results

**Figure 6:**
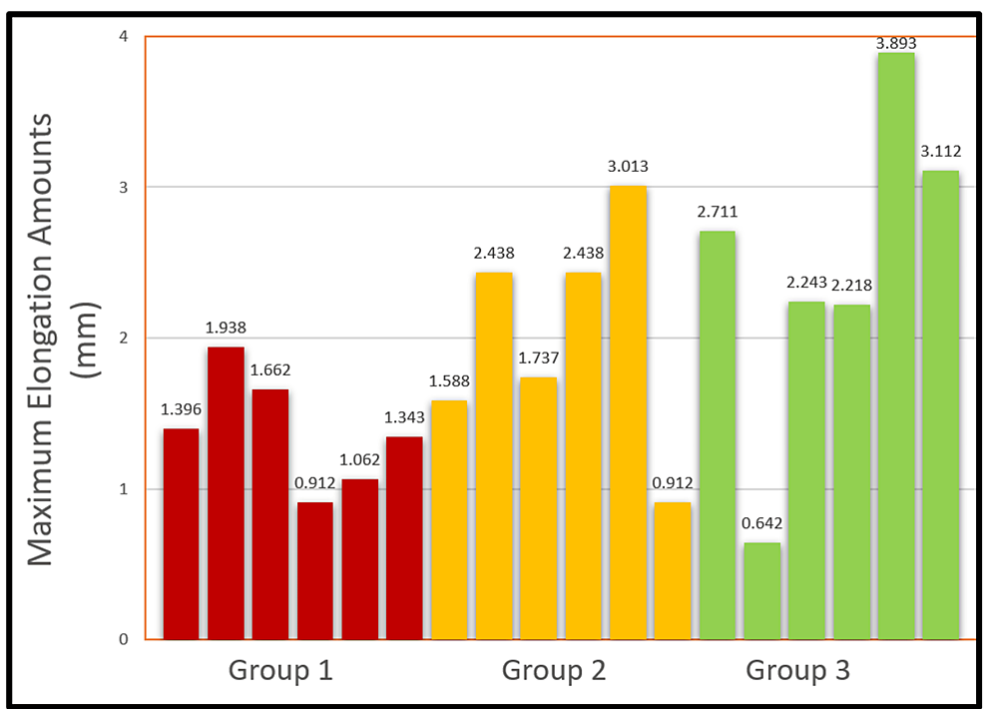
The maximum elongation amount of each sample in three groups.

The maximum elongation amounts in all samples subjected to biomechanical testing are shown graphically (Figure 6).

The mean MEA ± Standard Deviation (Range) was 1.39 ± 0.38 (0.91-1.94) for group 1, 2.02 ± 0.75 (0.91-3.01) for group 2, and 2.47 ± 1.09 (0.64-3.9) for group 3. There was no statistically significant difference between the groups regarding MEAs (p: 0.092).

**Figure 7:**
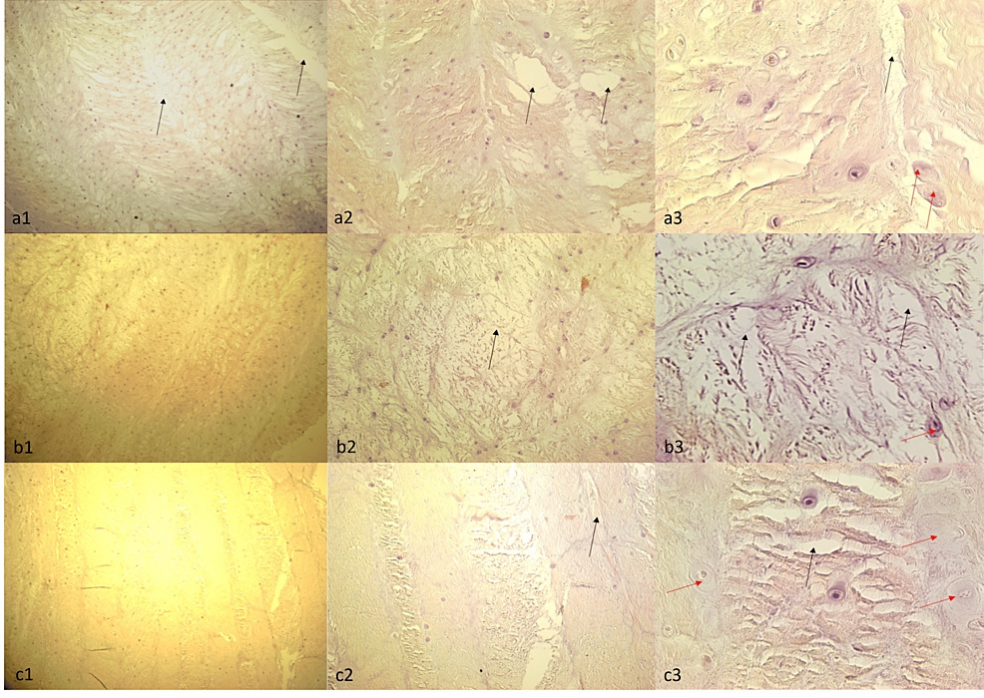
Hematoxylin & Eosin Staining of Group 1 (a1, a2, a3), Group 2 (b1, b2, b3), and Group 3 (c1, c2, c3) samples. The microscope magnifications used to examine the samples were 10x (a1, b1, c1), 40x (a2, b2, c2), and 100x (a3, b3, c3). Black arrows indicate connective tissue detachments and edema, and red arrows indicate necrotic cells.

Histological examination using Hematoxylin and Eosin staining revealed no statistically significant differences among the three groups (Group 1, Group 2, and Group 3). All groups exhibited similar pathological features, including cellularity, edema in the connective tissue, separation between tissue layers, delamination, and evidence of cellular swelling and necrosis (Figure 7).

**Figure 8:**
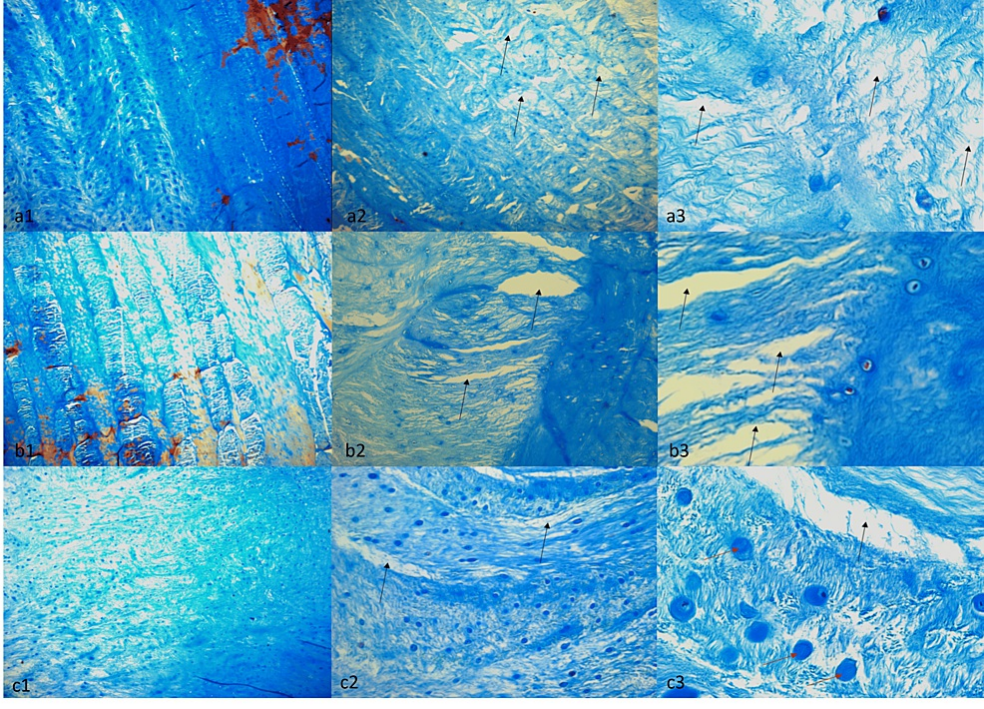
Alcian Blue Staining of Group 1 (a1, a2, a3), Group 2 (b1, b2, b3), and Group 3 (c1, c2, c3) samples. The microscope magnifications used to examine the samples were 10x (a1, b1, c1), 40x (a2, b2, c2), and 100x (a3, b3, c3). Black arrows indicate connective tissue detachments and edema, and red arrows indicate necrotic cells.

AB staining revealed a trend towards decreased GAG structure in the tissue samples, manifested as a somewhat weaker staining intensity relative to the control. Although no statistically significant differences were detected between groups, closer examination of AB staining revealed a slightly greater disorganization of the GAG-rich layers within Group 3's stratified structure (Figure 8).

**Figure 9:**
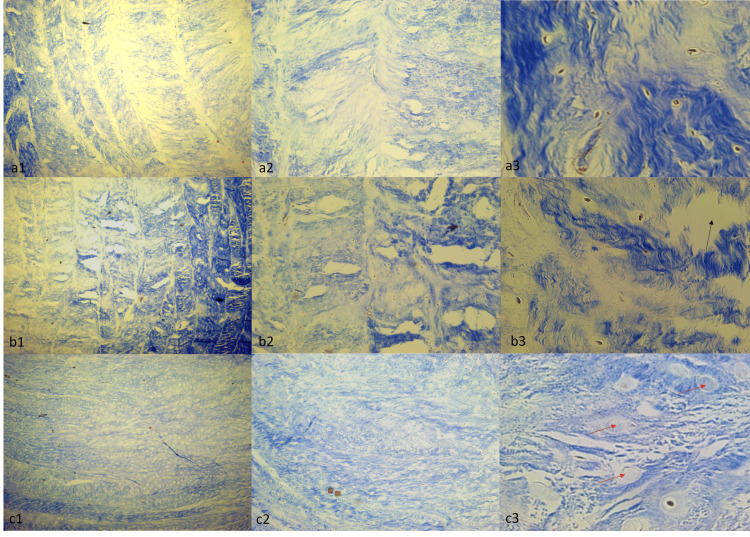
Masson’s Trichrome Staining of Group 1 (a1, a2, a3), Group 2 (b1, b2, b3), and Group 3 (c1, c2, c3) samples. The microscope magnifications used to examine the samples were 10x (a1, b1, c1), 40x (a2, b2, c2), and 100x (a3, b3, c3). Black arrows indicate connective tissue detachments and edema, and red arrows indicate necrotic cells.

Generally, deteriorations in collagen structure were observed in all groups after Masson's trichrome staining. It was observed that the amount of collagen was significantly reduced compared to the control tissue (Figure 9).

## Discussion

Back discomfort is a widespread issue that affects a significant number of people and leads to a reduction in workforce participation. Numerous studies have documented the elevated incidence of back complaints within the general populace, with an annual hospital admission rate of 15% in the adult population and a lifetime prevalence of 80% [1]. This health concern ranks among the leading causes of workforce and social activity loss, affecting 60%-90% of the adult population at some stage in their lives. Following upper respiratory tract diseases, chronic lower back pain is the second leading cause of workforce loss [3,4].

Up to 39% of cases of low back pain are attributed to intervertebral disc pathologies, according to clinical investigations. When examined with the subcategories of intervertebral disc pathologies, degenerative disc disease and lumbar disc herniation emerge as the most prominent [13].

The human spine provides movements under multidirectional, dynamic loads. To experimentally measure this response in vitro, excised human spinal segments have been extensively studied in mechanically uniaxial compression models to obtain the viscoelastic and poroelastic properties of the disc [16]. However, the loading of the spine in daily life is not limited to compression alone, so it is crucial to measure its behavior in all movements (flexion-extension, rotation, and axial compression).

In the current literature review, although there are studies on different loading models that the spine is exposed to in daily life, there are deficiencies in lumbar intervertebral disc histology after these loads. It is helpful to examine the literature in two different ways. First, biomechanical studies of the lumbar spine will be discussed.

Wilke et al., in their study using eight sheep vertebrae with a testing device that can bear load in a three-dimensional plane (six directions: axial compression, flexion-extension, lateral bending, anterior-posterior sliding, lateral sliding, and rotation), could not obtain disc herniation in every specimen. They observed herniation in four samples, protrusion in two samples, and delamination in two samples. Herniation and protrusion were documented visually, and delamination was found after examining the samples with an MRI. Not all of the spinal segments analyzed had the same intervertebral disc, and it was observed that the discs that developed herniation were the upper segment discs between L1-L2/L2-L3, except for one. They also stated that since they recorded video during the tests, the lesions in the study were seen within the first few cycles. They stated that their aim in this study was to create disc herniation by applying excessive loads, and that the same situation could be achieved by injecting increasing amounts of gel into the disc. In order to apply excessive load, the experiment was carried out with a torque of 7.5 NM, which was four times the fracture model study previously used by Wilke et al. [18]. UHF-MRI (ultra-high field MRI, 11.7 tesla), which showed delamination, was previously intended to show the changes in the sheep spine. As it would take approximately 10 hours to scan each sample due to the excessive rise in temperature during the application, only one sample was scanned with an MRI before the biomechanical test. The fact that more cartilage end plate damage occurred in the applied test (in four samples) than in discs suggested that the method caused more bone damage [19]. In our study, the biomechanical tests were based on the study protocol of Berger-Roscher N et al. in 2017 [17]. In our study, 800 N axial loading was applied to all spine samples, and the same segments (L2-L3 discs) of all samples were used for histological analysis. Since histological evaluation will be made, visible disc herniation was not deliberately created.

In another study by O'Connell et al., although the relationship between disc degeneration and disc mechanics is known, they planned to demonstrate the internal dynamics of the disc using MRI, citing the difficulty of examining disc tissue under mechanical loads without compromising tissue integrity. Twenty L3-L4 or L4-L5 samples obtained from 14 human cadaver vertebrae were potted with bone cement to form a functional spinal unit, and a 1000 N load was applied to each sample for 3 seconds in flexion, extension, and neutral positions. They found that the Annulus Fibrosus forces on the posterior side in the flexion position and the anterior side in the extension position were in favor of compression; in the same positions, the Annulus Fibrosus forces in the opposite region were tensile, and in the neutral state, the compressive forces in the posterior side were higher than the anterior and lateral regions. They stated that the 1000 N load they applied in their study would correspond to a moderate physiological load and found that the loss and displacement in disc height (0.65-0.55 mm) were similar to other studies. As a result, they stated that the tension in the posterior is greater with each load, but the physiological response may be different, which may change with degeneration. The study's limitation is that it applied motion in two-dimensional directions and that the initial value taken in stress measurement may have been high due to its small size [20]. In our study, we applied 800 N load, similar to this study, at a 30 mm/second speed.

In another study, Heuer and his colleagues applied simple and complex loadings to six human cadaver lumbar spines and created finite element models of the samples with sensors. They aimed to compare the intervertebral disc bulging and the annulus fibrosus stresses with simple and complex loading. The vertebrae were potted using bone cement from the proximal and distal ends, leaving the L2-L3 segments empty. Before the study, all samples were placed under a 500 N load for 15 minutes to reduce their water content. Then, simple loading with 500 N and other complex movements (combination of rotation, lateral bending, flexion, and extension movements) with 7.5 N/M torque were applied. The results were recorded. As a result, it was generally observed that there was more annulus fibrosus stretching and bulging with combined complex movements rather than simple loads. They found that herniation generally occurred in the posterolateral part of the specimens [6].

If we talk about the histology of the lumbar spine, it would be helpful to look at the study of Roberts et al. in 2006. This study mentioned that the disc undergoes degenerative changes in the early stages of life. The decrease in the number of cells in the annulus fibrosus and the changes in the cartilage endplate may contribute to this. In human cadaver histology studies, it has been shown that the number of vessels in the cartilage endplate gradually decreases after 30 months. It is thought that with aging, there will be a decrease in cell proliferation and clustering and an increase in cell death (apoptosis), and increased cytokines and matrix metalloproteinases will cause this. It has been shown that the structure and distribution of matrix molecules such as collagen, elastin, and fibronectin will change, and the structure and amount of glycosaminoglycans will decrease. As a result, it has been stated that the mechanical and physiological functions of tissues may play a role in the pathogenesis of these disorders [21].

In a similar study, Boos et al. made macroscopic and histological evaluations of 180 human cadaver spines. In their research, they tried to develop a histological classification system instead of the traditional macroscopic assessment of disc herniation. They found that their classification was correlated with the macroscopic classification. Their study stated that the basis of macroscopic disc changes began to be demonstrated histologically after the second decade of life [22].

The study of Berger-Roscher et al., which was taken as the basis for our research, aimed to create failure in lumbar spine specimens where four-way movement was applied (flexion, lateral bending, rotation, and axial compression). Thirty functional spinal units obtained from nine sheep vertebrae were potted using bone cement to perform the test. The loads were applied under 800 Newton axial compression, with 13 degrees of flexion, 10 degrees of lateral bending, and 4 degrees of rotation. After the biomechanical test, an MRI was performed on the materials. As a result, cartilage end plate damage developed in 17 of them, 13 large and four small, but no visible herniation occurred in any of the samples. In the study's specimens, 76% of the cartilage endplate fractures and 24% of the anulus fibrosus damage were observed. They concluded that the highest risk for cartilage end plate damage was when all movements were performed together [17].

We tried to see whether there were histological and biomechanical differences between the sheep vertebrae we used in our study by applying loads in three different ways. After biomechanical testing, we appropriately removed the intervertebral discs and preserved them for histology. We similarly cut one disc of material without applying stress and reserved it for baseline histological evaluation for control purposes. The maximum amount of elongation obtained after the study was achieved in the group (Group 3), in which rotation, flexion, and axial rotation were applied thanks to the particular device. However, after statistical analysis, the difference was not significant. The maximum amount of elongation increased from Group 1 to Group 3. Since this indicates increasing displacement depending on the type of loading applied, it may indicate that the disc, facet joint, and ligament structures are under more stress than the bone structures in in-vivo conditions. Increased displacement may indicate a greater possibility of herniation during combined movements. However, in vivo studies are needed to demonstrate this clearly.

After the histological evaluation, no difference was detected between the groups in Hematoxylin and Eosin staining. However, edema in the connective tissue, separations between tissue layers, and layering deteriorations were observed. After staining with Alcian blue, there was a decrease in the glycosaminoglycan structure in the cartilage tissue compared to the control tissue, deteriorations in the structural arrangement were observed, and it is noteworthy that the layering was disrupted in Group 3. These findings may suggest that lumbar disc herniation may be associated with axial loading along with rotation, as this means that the deterioration in the annulus fibrosus tissue will be more significant with rotation. Again, similar findings with Masson's trichrome staining support our idea.

The main limitations of our study are the possible differences between human lumbar intervertebral discs since it was a sheep spine study. Although MRI is frequently used in diagnosing and following disc herniation, the fact that MRI imaging was not performed after biomechanical testing in this study is another limiting factor. Not measuring intradiscal pressures can be considered another limitation. Also, the fact that finite element analysis was not performed by measuring intra-discal and cartilage end plate tensions can be regarded as a limitation. Since the test in this study was conducted in a biomechanical laboratory environment, this method does not fully reflect the effects of functional anatomy and in vivo conditions. Another deficiency of the study is that since there are no soft tissues around the spine, the impact of these soft tissues on the bones, discs, and ligaments, in short, on the spine, cannot be fully simulated.

## Conclusions

Lumbar disc herniation is one of the leading causes of low back pain. The multidirectional movements of the mobile segments of the human lumbar spine make it challenging to study the mechanisms of disc herniation formation. Biomechanically, when multidirectional loading is applied, the vertebrae's higher displacement may indicate that the disc, facet joint, ligament structures, and bone structures are under more stress in in-vivo conditions. Increased displacement may suggest that herniation is more likely to occur during combined movements.

In addition, after histological studies, the fact that the deterioration in the layering is less in the discs given only axial load, the deterioration between the tissue layers is more significant with complex movements, and the deterioration of the layering is greater than other loading combinations may indicate the role of the complex loads in the formation of disc herniation. More specific findings can be obtained by furthering our study with human cadaver models by including the degenerative process in the spine.
